# Orangutans (*Pongo abelii*) make flexible decisions relative to reward quality and tool functionality in a multi-dimensional tool-use task

**DOI:** 10.1371/journal.pone.0211031

**Published:** 2019-02-13

**Authors:** Isabelle B. Laumer, Alice M. I. Auersperg, Thomas Bugnyar, Josep Call

**Affiliations:** 1 Department of Cognitive Biology, University of Vienna, Vienna, Austria; 2 Messerli Research Institute, University of Veterinary Medicine, Vienna, Medical University of Vienna, University of Vienna, Vienna, Austria; 3 School of Psychology and Neuroscience, University of St. Andrews, St. Andrews, United Kingdom; 4 Max Planck Institute for Evolutionary Anthropology, Leipzig, Germany; Texas A&M University, UNITED STATES

## Abstract

Making economic decisions in a natural foraging situation that involves the use of tools may require an animal to consider more levels of relational complexity than merely deciding between an immediate and a delayed food option. We used the same method previously used with Goffin´s cockatoos to investigate the orangutans’ flexibility for making the most profitable decisions when confronted with five different settings that included one or two different apparatuses, two different tools and two food items (one more preferred than the other). We found that orangutans made profitable decisions relative to reward quality, when the task required the subjects to select a tool over an immediately accessible food reward. Furthermore, most subjects were sensitive to work-effort when the immediate and the delayed option (directly accessible by using a tool) led to the same outcome. Most subjects continued to make profitable decisions that required taking into account the tool functionality. In a final multidimensional task design in which subjects had to simultaneously focus on two apparatuses, two reward qualities and two different tools, the orangutans chose the functional tool to access the high quality reward.

## Introduction

Making rational economic decisions in natural foraging situations requires balancing costs (such as time spent to travel to a feeding patch) and benefits (such as gaining food of higher quality). Moreover, such decisions often require individuals to consider several factors simultaneously including the predictability of finding a more valued/ ripe food source, the reachability of the food source as well as the presence of the proper means /tools to open up an extractable food.

The aim of this study was to investigate how orangutans´ decision-making processes are affected by tool-use. Orangutans are the largest predominantly arboreal frugivores that regularly use tools in the wild and captivity (e.g. [1–4]). Nevertheless, in their natural habitat, such as the Bornean peatland forest of Central Kalimantan which is home to the largest population of orangutans in Indonesia [5], fruits are distributed in patches and are available in ripe form only during a rather short, unpredictable and fluctuating period of time. In extreme cases Bornean orangutans have to face periods with only <3% of trees fruiting for durations up to almost one year. Optimality models [6] predict that orangutans should adopt different foraging strategies depending on the fruit availability: When high quality food is scarce (such as fruits), they will react either by including lesser-quality food into their diet (bark and leaves) or by travelling further to find a high quality food patch [7]. Thus, testing orangutans in a controlled setting allows us to better understand their natural tendencies and decision-making processes.

The ability to forego an immediate reward for the prospect of receiving a delayed but qualitatively or quantitative better option for more than a couple of seconds has so far only been found in orangutans as well as several other large brained animals, like some other primate species, dogs, corvids and parrots (e.g. [8–15]). Interestingly there are only a few primate studies that included the effect of tool use on a delayed decision-making process in their task design: Tufted capuchin monkeys inhibited eating a rod-shaped piece of celery in order to use it as a tool to push peanut butter out of a tube. In contrast, all subjects immediately ate the piece of celery when the tube was unbaited [16]. Furthermore tufted capuchin monkeys exchanged a piece of food for a tool only when they could use it to get access to a higher quality food [17]. When given the choice between a likable food reward (a grape) and a straw tool, chimpanzees and an orangutan chose the tool if they could use it 70 minutes later to sip the more preferred fruit soup [18]. Thus, it seems that at least one orangutan and some other primate species can inhibit eating an immediate food reward for the prospect of getting another food item of a higher quality by using a tool, even if this involves waiting for more than one hour [18].

In order to gain a better understanding of the different aspects of orangutans´ decision-making processes, we used a standardized method that was recently used on a bird species, the Goffin’s cockatoo (*Cacatua goffiniana*). Although these birds are not habitual tool users in the wild, they have proved to be skilled tool users in captivity [19]. Both Goffin cockatoos and orangutans are opportunistic generalists that incorporate a large variety of fruits into their diet that require extractive foraging [20, 21]. Furthermore both are largely arboreal and live in evergreen Indonesian tropical rain forests characterized by high annual fluctuation of flower and fruit production [22]. Therefore, both animals might face similar ecological pressures in terms of finding and exploiting fruit patches, making optimal decisions in terms of weighing costs and benefits of staying or moving to find another high quality food patch.

The Goffin cockatoos were tested in five different setups that included two different apparatuses and their respective tools and two food qualities. When required to make decisions between using a tool in order to access a food reward inside an apparatus or eating an immediate food reward, Goffin cockatoos were able to make profitable decisions relative to reward quality, tool functionality and work effort [19]. Nevertheless, as task complexity increased (all task components, two tools, two apparatuses and two food qualites were present), Goffin cockatoos were not able to focus on the profitable option anymore. A detailed study on the trap-tube-problem in chimpanzees, bonobos and orangutans showed that when subjects were required to process two spatial object-object relations simultaneously (here: tool-reward and reward-surface relation) their task success deteriorated, presumably because their cognitive resources were overtaxed [23]. Therefore, it would be interesting to test whether a habitual tool using ape species would be able to choose the profitable option in this tool-use task.

Orangutans should be able to make profitable decisions relative to reward quality since they were able to inhibit eating preferred food for more than one minute in order to maximize their profit [9] and one orangutan was even able to choose a tool over an immediate preferred food item if she could access a more preferred food by using the tool [18]. Furthermore they should be able to integrate the factor tool functionality into their decision-making process, since they proved to be able to select a functional tool based on rigidity, length and diameter [24, 25] and were able to use up to five tools sequentially in order to obtain a functional tool [26]. Thus, we expected them to make profitable decisions in regard to the functionality of the available tool and at the same time to be able to choose a tool over an immediate food item to obtain a higher quality food reward. Since the ability of Goffin's cockatoos to maximize their profit was constrained when the complexity of the task increased and orangutans’ performance was negatively affected when they were required to process two spatial relations simultaneously [23], it was unclear whether orangutans would increase their pay-off in this task.

## Methods

### Subjects and housing

Six orangutans participated in this study, four adult females, one adult male and one juvenile male (S1 Table). All subjects were born in captivity and were housed in a social group in a large, enriched indoor (approx. 200m^2^) and outdoor enclosure (approx. 1800m^2^) at the Wolfgang Köhler Primate Research Center in Zoo Leipzig, Germany. The enclosures were equipped with various enrichment items and natural climbing structures. Food and water were available ad libitum. During the three main meals, the orangutans received fresh fruits, vegetables, eggs, cereals, leaves and sometimes meat. All experiments were non-invasive and based purely on behavioural tests. The research was approved by the ethics joint committee of the Max Planck Institute for Evolutionary Anthropology and Zoo Leipzig and the methods were carried out in accordance with the relevant guidelines and regulations. Animal husbandry and research comply with the EAZA minimum standards for the accommodation and care of animals in zoos and aquaria and the WAZA ethical guidelines for the conduct of research on animals by zoos and aquariums. The research adhered to all German laws regarding animal testing and holding. All orangutans participated on a voluntary basis, were and were never food or water deprived and returned after testing to their enclosure. All orangutans were tested individually. Prior to this study subjects had participated in a variety of non-invasive cognitive tasks. All apes had experience in inserting compact and stick tools and some of them had experience using the ball- and stick-apparatus (unpublished data) but not in the presented context.

### Apparatuses and training

**Fig 1:**
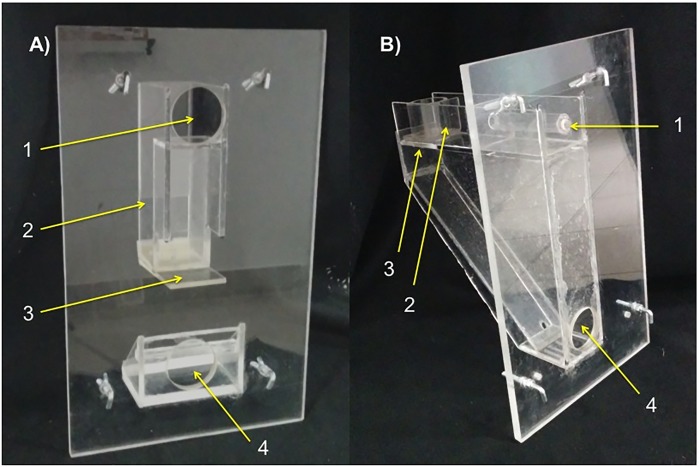
**Left:** A) Ball-apparatus 1) Insertion hole 2) vertical tube shaft 3) position of food reward on collapsible platform 4) reward opening. **Right:** B) Stick-apparatus 1) Insertion hole 2) moveable tube-cup (open at the bottom), food reward is located inside of it 3) opening to slanted panel 4) reward opening.

We used two apparatuses (ball, stick) and their respective tools (ball, stick) (see Fig 1). Each apparatus could only be operated with their corresponding tool. The apparatuses were similar to the ones used in the Goffin cockatoo study [19]. The ball-apparatus consisted of a transparent box whose interior contained with a collapsible platform upon which the food item rested on. When the ball tool was inserted, it fell through a vertical tube, it hit and collapsed the platform and releasing the food item (see Fig 1). The stick-apparatus was a transparent box with a smaller opening (ca. 15 mm; only the stick could be inserted). The food reward rested inside a movable transparent cup. By inserting the stick tool the cup could be pushed towards the opening to a slanted platform, thereby releasing the food (see Fig 1).

Prior to testing subjects received a total of 40 training trials with each apparatus (stick- and ball-apparatus), in which they were given the functional tool and successfully operated the respective apparatus. Furthermore, subjects also experienced that only one tool was functional in the corresponding apparatus (for more details see SI, chapter B).

### Testing procedure and design

A single apparatus was attached to the mesh panel (semi-randomly balanced across sessions for each testing condition) in all tests except in the TSQAT in which both the stick and ball apparatuses were attached to the mesh. The experimenter (IBL) baited the apparatus/es in front of the subjects and immediately placed two items on a sliding table. Depending on the test/condition, the orangutans faced binary choices between two tools, or a tool and a food item. Subjects signalled their choice by pointing with the finger. After three seconds (in the *TSQAT* 9 seconds) the experimenter moved the sliding table towards the mesh and the subject could select one of the two items on the platform. The other item was immediately removed. If subjects chose the non-functional tool they had to wait in front of the apparatus for 30 seconds until the next trial began. The side of presentation was semi-randomly balanced across sessions for all conditions. During testing the experimenter wore mirrored sunglasses, avoided any head movements, and remained silent.

Prior to testing, we assessed subjects’ preferences for highly desirable food items that were usually always eaten straight away (apple, grape, banana-pellet). For the test we used only two of these foods. The high quality food had to be chosen over the lower food quality in at least 80% of binary choices. We used subject´s most preferred food as the high quality food and the third preferred food as the lower quality food in the test. All orangutans chose the banana-flavoured pellet (high quality) over the apple (lower quality) in more than 90% of binary choices. For the adult male we used grape as lower quality and banana-pellet as high quality food. To control for potential preference changes during testing, we re-assessed food preferences during and at the end of the testing phase. Food preferences remained unchanged (S2 Table).

**Fig 2:**
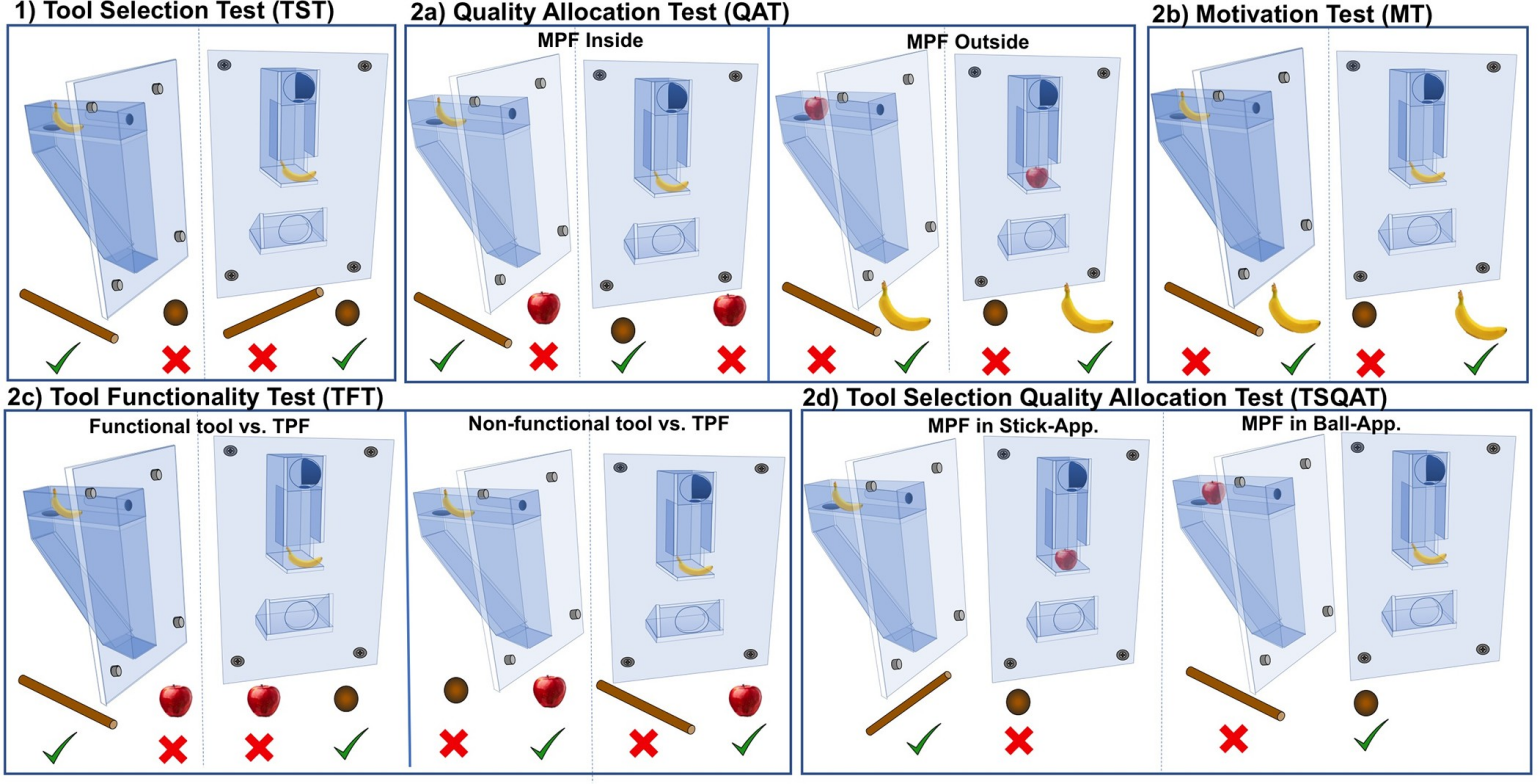
Overview of the conditions within each of the five tests. High quality food is banana-pellet, lower quality food is apple. 1) TST (Tool selection test): both tools are present while the high quality food is inside the apparatus. 2a) QAT (Quality allocation test): the tool is functional in each of the conditions. Left: High quality food is inside the apparatus. Immediate food option is the lower quality food. Right: Lower quality food is inside the apparatus. The high quality food is the immediate food option. 2b) MT (Motivation test): tool is always functional. High quality food is inside and outside the apparatus. 2c) TFT (Tool functionality test): High quality food is inside the apparatus in each condition. Left: Functional tool is present. Right: Non-functional tool is present. 2d) TSQAT (Tool selection quality allocation test): both tools and both apparatuses are present. Left: High quality food is located in the stick-apparatus. Right: High quality food is located in the ball-apparatus.

Due to the small sample size of available orangutans, which prevented us from conducting statistical group comparisons, we kept the same order of the tests for all subjects (note that in the Goffin cockatoos there was no difference in performance in the order in which the tests were conducted). Thus, subjects received the five tests in the following order: Tool selection test (*TST*), Quality allocation test (*QAT*), Motivation test (*MT*), Tool functionality test (*TFT*) and Tool selection/quality allocation test (*TSQAT*; for an overview of all tests see Fig 2 and detailed description of all tests below).

### 1) Tool selection test (TST)

This test assessed subjects´ knowledge about the functionality of the tools. The apparatus (stick- or ball-apparatus) always contained the subject´s high quality food (see Fig 2. 1). The orangutans had to choose between the ball and the stick tool. Although we only analyzed the first 12 trials in each condition, subjects received as many 12-trial sessions as needed until they reached the criterion of selecting the correct tool in at least 83% of the trials in two consecutive sessions. We did this to ensure that all subjects possessed the prerequisite knowledge needed for subsequent tests.

### 2a) Quality allocation test (QAT)

This test investigated whether subjects responded flexibly by choosing either the tool or the food item depending on the location of the high quality food. There were two conditions: the high quality food was either inside or outside the apparatus (see Fig 2. 2a). Thus, the apparatus contained either the subject´s high or lower quality food (in 50% of the trials, semi-randomly balanced). Depending on the condition, subjects had to decide between the tool and the lower quality food or the tool and the high quality food. The combination of the high quality food (inside or outside of the apparatus) and type of apparatus (ball- or stick-apparatus) generated four possible conditions that we presented in a semi-randomized fashion across four sessions. Each orangutan received a total of four 12-trial sessions.

### 2b) Motivation test (MT)

Th*is test* tested whether subjects had acquired a preference for either the immediate food or the food inside the apparatus. There were two conditions (stick- and ball-apparatus condition) that were presented in a semi-randomized order across sessions (see Fig 2. 2b). Either the stick- or the ball-apparatus was attached to the mesh. The high quality food was located inside as well as outside of the apparatus. The tool was always functional. The orangutans had to choose between the immediate food reward or the tool. Subjects received a total of two 12-trial sessions.

### 2c) Tool functionality test (TFT)

This test investigated whether subjects could maximize their profit by correctly assessing the functionality of the present tool. We administered two conditions that were presented in a semi-randomized order across sessions (functional vs. non-functional tool condition; see Fig 2. 2c). The apparatus (stick- or ball-apparatus) contained the high quality food reward in all trials while the orangutans could choose between the lower quality food reward and depending on the condition either a functional or non-functional tool. The tools were functional in 50% of trials and the trials were semi-randomly balanced across sessions. The combination of type of apparatus (stick- or ball-apparatus) and the type of tool (functional vs non-functional) generated four possible conditions that we presented in a semi-randomized fashion across four sessions. Each orangutan received a total of four 12-trial sessions.

### 2d) Tool selection/quality allocation test (TSQAT)

This test investigated whether subjects could maximize their profit when all task components were present (two apparatuses, two tools, two food qualities). Both apparatuses were attached to the mesh panel. The side of presentation was semi-randomly balanced across sessions (see Fig 2. 2d). We set up two possible conditions in which the high quality food was either located inside the stick- or ball-apparatus while the other apparatus was baited with the lower quality. The orangutans had to choose between the stick- or ball-tool. After 15 seconds of apparatus inspection, both tools were placed on the platform. After nine seconds subjects could make their choice. The *TSQAT* comprised two 12-trial sessions.

### Analysis

All data was HD video recorded and coded in situ as well as from the videos. As the data did not meet the criteria for parametric analysis we used non-parametric two-tailed statistics. Statistical tests were conducted in IBM SPSS using exact procedures. Further T^+^ values were calculated and if required the p-values were adjusted according to recommended procedures for small sample sizes [27]. To investigate a possible learning effect we ran a pairwise comparison (Wilcoxon signed rank tests, p<0.05) for the first and last six trials of each condition for each test. Although testing was carried out until criterion in the *Tool selection test*, only performance in the first two sessions was analysed.

## Results

### 1) Pretest tool selection test (TST)

Fig 3.1 presents the percent of trials in which orangutans selected the stick tool (over the ball) as a function of the type of apparatus. Orangutans selected the stick tool significantly more often when facing the stick apparatus than the ball apparatus (Wilcoxon test: T^+^ = 21, p = 0.031). Analyses at the individual level confirmed this result for four of the six individuals (Fisher test: P<0.01, S3 Table). Five and three of the six orangutans significantly chose the stick tool and ball tool above chance (Binomial test: p<0.05) in the stick and ball apparatus, respectively (S3 Table). The three subjects who did not initially reach the criterion (Dokana, Suaq, Bimbo) needed up to seven sessions to reach it (S3 Table).

**Fig 3:**
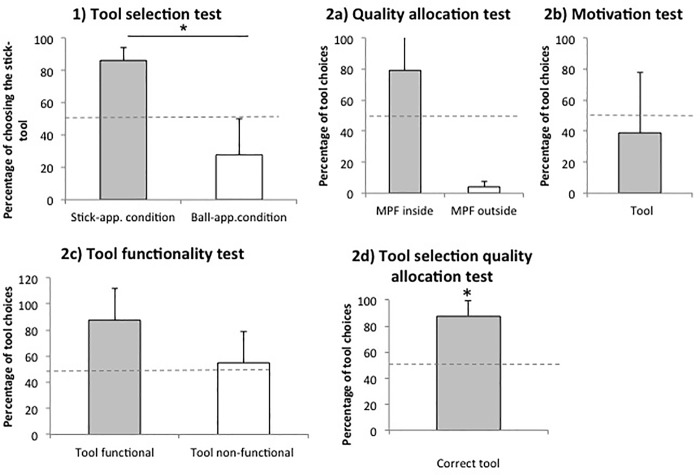
Mean percentages of tool choices per condition within each test. 1) *TST (Tool selection test)*, 2a) *MT (Motivation test)*, 2b) *QAT (Quality allocation test)*, 2c) *TFT (Tool functionality test)*, 2d) *TSQAT (Tool selection quality allocation test)*; (n = 6). * p<0.05.

The orangutans’ performance did not significantly differ between the stick- and ball-apparatus condition in any of the tests (Wilcoxon tests, all p>0.05 in all cases; S8 Table). Furthermore there were no learning effects detectable when comparing the first six trials to the last six trials of each test (Wilcoxon signed rank tests, all p>0.05, S9 Table). Consequently, we collapsed the data with regard to apparatus type and trial number in subsequent analyses.

### 2a) Quality allocation test (QAT)

Fig 3.2a presents the percent of trials in which orangutans picked the tool as a function of the high quality food’s location. Orangutans did not select the tool significantly more often when the high quality reward was inside compared to outside the apparatus (Wilcoxon test: T^+^ = 20, p = 0.063). However, this result was caused by Bimbo who selected the tool only once (out of 24 trials) when the high quality food was outside the apparatus. Individual analyses confirmed that all other five orangutans selected the tool significantly more often when the high quality reward was inside compared to outside the apparatus (Fisher´s exact tests: all p<0.001, S4 Table).

### 2b) Motivation test (MT)

Fig 3.2b presents the percentage of trials in which orangutans chose the tool over the immediate food option when the food outside and inside the apparatus was of the same quality (high quality). Orangutans did not select the tool over the immediate food option above chance level (Wilcoxon test: T^+^ = 12, p = 0.844). On an individual level four orangutans significantly chose the immediate food option over the tool, whereas two individuals significantly chose the tool over the immediate food reward (Binomial test; all p<0.05; S5 Table).

### 2c) Tool functionality test (TFT)

Fig 3.2c presents the percentage of trials in which the orangutans selected the functional tool (over the lower quality food) and the lower quality reward (over the non-functional tool). Orangutans did not select the tool significantly more often when the tool was functional or non-functional (Wilcoxon test: T^+^ = 15; p = 0.063). On an individual level four out of the six individuals selected the tool more often when it was functional (Fisher´s exact tests: all p<0.05; S6 Table).

### 2d) Tool selection quality allocation test (TSQAT)

Fig 3.2d presents the percentage of trials in which the orangutans selected the correct tool as a function of the high quality food´s location (either in the stick- or ball-apparatus). Subjects did select the correct tool above chance level (Wilcoxon test: T^+^ = 21, p = 0.031). On an individual level five orangutans chose the profitable tool above chance levels (Binomial tests: all p<0.01; S7 Table).

## Discussion

Orangutans maximized the quality of their food intake by flexibly choosing between a food item and a tool depending on the conditions. In particular, they chose a tool over an immediately accessible food item to obtain a more preferred food item (using the tool) but they chose the immediate food item over the tool if the apparatus contained the food item of a lower quality (*Quality allocation test*). Additionally, the majority of subjects showed work-effort sensitivity by choosing the immediate food option over the tool if the food inside the apparatus was of the same quality (*Motivation test*). Four of the six orangutans made profitable decisions when the task required the subjects to simultaneously focus on the functionality of the tool as well as on the reward quality (*Tool functionality test*). Furthermore, when subjects faced both apparatuses (each baited with food items that differed in quality) and both tools (*Tool selection quality allocation test*), all orangutans chose the functional tool to operate the apparatus containing the higher quality food.

After subjects consistently selected the appropriate tools in the pretest (Tool selection test), we found no evidence that subjects learned to select the correct alternatives over the course of the testing phase. Prior to the test phase, two males and a female needed some training to reach above performance in the ball apparatus. A possible explanation for this difference between apparatuses is that the orangutans were, prior to this experiment, tested in several tasks requiring the use of sticks but not the insertion of balls (e.g. [25]). The other three subjects immediately reached the criterion for both the stick- and ball-apparatus condition, which is consistent with studies in great apes and capuchin monkeys that selected tools based on their properties [25, 28–31].

In the *Quality allocation test*, all subjects except Bimbo flexibly chose the profitable option in all test conditions. These findings are consistent with the results of previous primate studies, although these studies only investigated certain aspects of our *Quality allocation test*. More specifically, tufted capuchin monkeys used a lower quality food as a tool to obtain high quality food from a tube if it was baited [16] and they only exchanged a lower quality food for a tool if they could access a higher quality food with it [17]. Moreover, chimpanzees and one orangutan selected a tool over an immediate food item if they could use it to obtain a higher quality food 70 minutes later [18]. On an individual level ten out of 13 Goffin cockatoos that were tested in the *Quality allocation test* were able to choose the profitable option (data of stick and ball apparatus pooled, note that in the original paper by Laumer et al. 2016 the data was not pooled; One Goffin was able to be successful above chance expectation in all test conditions of the *Quality allocation test*).

Optimality models [6] predict that orangutans should adopt different foraging strategies depending on the fruit availability: When high quality food is scarce (such as fruits), they will react either by including lesser-quality food into their diet (bark and leaves) or by travelling further to find a high quality food patch [7].

Similar to the Goffin cockatoos, the majority of subjects chose the immediate food option in the *Motivation test* when the reward inside and outside of the apparatus was of the same quality (high quality). Only two females (Pini and Raja) chose the tool over the immediately accessible high quality food. It is conceivable that the choices of those two females (and those of the Goffin cockatoos that chose similarly), do not necessarily reflect a lack of understanding or efficiency but they might simply reflect a preference for using tools.

However, such preference has its limits as evidenced by the results of the *Quality allocation test*. Here the same two orangutans “sacrificed” the tools and instead chose a food item which quality was higher than the quality of the item that they would have obtained using the tool.

In the *Tool functionality test* four of the six orangutans simultaneously considered the functionality of the tool as well as the reward quality. This was particularly clear in the case of Raja, although she did not perform above chance level in both conditions of the ball-apparatus condition. Besides Raja, three other orangutans (Dokana, Padana and Suaq) chose the tool (as opposed to the lower quality food item) more often when it was functional, thus confirming apes’ and capuchin monkeys’ appreciation of tool functionality even in the absence of trial-and-error [28–29]. However, unlike those other studies, orangutans in the present study still selected the non-functional tool in a sizable number of trials. In contrast, three of the cockatoos were able to select the correct option in both conditions when confronted with the stick-apparatus and two of them were able to successfully maximize their profit in all conditions of the *Tool functionality test* [19]. It is possible that most apes failed to inhibit choosing a tool in this task due to a strong quality difference between apple (low quality) and the banana-flavoured pellet (high quality). In cockatoos two different types of nuts were used (pecan and cashew) and the quality gap between the two was arguably smaller. If that was the case, it is possible that the orangutans might rather accept losing the apple piece in order to try to operate the incompatible apparatus. Note also that they inserted the stick-tools into the ball-apparatus in 72% of failed trials. They may have experienced successes with stick tools through persistence in previous studies (e.g. [32–34]). Since none of the subjects was ever successful at doing that, they began to choose the apple piece more often when the tool was non-functional in order to avoid obtaining nothing at all.

In contrast to the parrots that were unable to focus on the profitable option in the *Tool selection quality allocation test* [19], all four female orangutans performed significantly above chance level by choosing the correct tool to access the high quality food. This result is consistent with a study showing that apes were able to select suitable tools for three different apparatuses presented sequentially in different days [25]. Unlike that study, however, orangutans in the current study had to select one of two tools to use in one of two baited apparatuses that were presented simultaneously. Keeping track of multiple tools and multiple baited apparatuses may have caused the difficulties that the male orangutans experienced here. Although they selected the stick when the high quality food was in the stick-apparatus above chance level, Bimbo only tended to select the ball-tool when the ball-apparatus was baited with the high quality food. The subadult (seven-year-old) Suaq chose the profitable option only at chance level (note that it took him already seven sessions to reach the criterion for the ball-apparatus in the pretest). Völter and Call (2014) found that the performance of chimpanzees, bonobos and orangutans dropped when facing a trap-tube problem that required the subjects to simultaneously consider two spatial object-object relations (tool-reward and reward-surface relation). The authors suggested that this failure might be due to a cognitive overload in the attentional system. Nevertheless, in the present study the multidimensional task design that required the subjects to simultaneously focus on two apparatuses, two reward qualities and two different tools seemed to fall below the orangutans limit in information processing.

Taken together the orangutans were able to make profitable decisions relative to reward quality and the use of a tool and even considered the additional work-effort of using a tool. Although orangutans previously showed problems when processing two spatial relations at the same time in another task design [24], subjects could maximize their profit in a multi-dimensional task that required them to take into account the apparatuses, the food items, and the tools. Future studies could systematically investigate orangutans´ limits of information processing by including more levels of relational complexity. Furthermore in order to investigate the mechanisms of decision-making relative to the use of tools into further detail, applying present task design in other large-brained human and non-human habitually tool-using species would be highly desirable.

## Supporting information

S1 Table Names, sex and year of birth and rearing history of the six orangutans (*Pongo abelii*). (PDF)

S2 Table Results of the preference tests in percent (%) including all combinations (a = apple, g = grape, p = banana pellet, r = rusk; *TPF* = third preferred food, *MPF* = most preferred food). Preference test 1 was conducted before subjects entered the test, Preference test 4 was conducted after all subjects had received all test trials. Preference test 2 &3 was only conducted with Bimbo due to reasons explained above.
(PDF)

S3 Table Number of correct trials out of a total of 12 trials for each condition in the *TST* for each individual. Binomial probabilities: * = p<0.05 (10/12 correct), ** = p<0.01 (11/12 correct); *** = p<0.001 (12/12 correct).
(PDF)

S4 Table Number of correct trials out of a total of 12 trials for each condition in the *QAT* for each individual. Binomial probabilities: * = p<0.05 (10/12 correct), ** = p<0.01 (11/12 correct); *** = p<0.001 (12/12 correct).
(PDF)

S5 Table Number of correct trials out of a total of 12 trials for each condition in the *MT* for each individual. Binomial probabilities: * = p<0.05 (10/12 correct), ** = p<0.01 (11/12 correct); *** = p<0.001 (12/12 correct).
(PDF)

S6 Table Number of correct trials out of a total of 12 trials for each condition in the *TFT* for each individual. Binomial probabilities: * = p<0.05 (10/12 correct), ** = p<0.01 (11/12 correct); *** = p<0.001 (12/12 correct).
(PDF)

S7 Table Number of correct trials out of a total of 12 trials for each condition in the *TSQAT* for each individual. Binomial probabilities: * = p<0.05 (10/12 correct), ** = p<0.01 (11/12 correct); *** = p<0.001 (12/12 correct).
(PDF)

S8 Table Results of the paired Wilcoxon tests for subjects´ performance in the ball- and stick- apparatus condition for each condition and for each test (n = 6). (PDF)

S9 Table Results of the paired Wilcoxon tests for the first and last six trials of each condition for each test (n = 6). (PDF)

S1 Movie Movie of the Tool selection test (*TST*), Motivation test (*MT*), Quality allocation test (*QAT*), Tool functionality test (*TFT*) and Tool selection quality allocation test (*TSQAT*). (MOV)
