# Supplementary material for: Orangutans (Pongo abelii) make flexible decisions relative to reward quality and tool functionality in a multi-dimensional tool-use task
Source: PLoS One. 2019 Feb 13;14(2):e0211031. doi: 10.1371/journal.pone.0211031 (PMC6374006; doi:10.1371/journal.pone.0211031)
Supplement: S1 Table — (PDF) [file pone.0211031.s001.pdf]

S1 Table Names, sex and year of birth and rearing history of the six orangutans (*Pongo abelii*).

Name	Sex	Date of birth	Rearing
Bimbo	male	20.09.1980	hand reared
Dokana	female	31.01.1989	mother reared
Padana	female	18.11.1997	mother reared
Pini	female	30.06.1988	mother reared
Raja	female	26.09.2003	mother reared
Suaq	male	14.05.2009	mother reared

Training phase

Methods

Prior testing subjects received a total of 40 training trials with each apparatus (stick- and ball-apparatus), in which they were given the functional tool and successfully operated the respective apparatus.

To give the orangutans the experience that each tool is a functional key to one but not to both apparatuses, subjects received 50 trials in which they were confronted with either the stick- or the ball-apparatus (semi-randomly mixed) and were given the respective non-functional tool. After 20 seconds subjects were signaled to give back the tool and were given the functional tool. If subjects dropped the non-functional tool out of the testing compartment during the 20s time-interval, it was replaced by the functional tool.

To ensure that subjects paid attention to the apparatuses´ food content, two sessions of 12 trials were conducted in which the apparatus contained either subject´s *TPF* or *MPF* (semi-randomly mixed).
