# Supplementary material for: Orangutans (Pongo abelii) make flexible decisions relative to reward quality and tool functionality in a multi-dimensional tool-use task
Source: PLoS One. 2019 Feb 13;14(2):e0211031. doi: 10.1371/journal.pone.0211031 (PMC6374006; doi:10.1371/journal.pone.0211031)
Supplement: S2 Table — Preference test 1 was conducted before subjects entered the test, Preference test 4 was conducted after all subjects had received all test trials. Preference test 2 &3 was only conducted with Bimbo due to reasons explained above. (PDF) [file pone.0211031.s002.pdf]

Qualitative preference test

Food preferences for three desirable food types (apple, grape, banana-pellet) were identified (see Table S2). We used the most preferred food as high quality food in the test and the third preferred food as lower quality food. In order to control for possible preference changes, preferences for subject´s most preferred food (*MPF*) and third preferred food (*TPF*) were retested during the testing phase. We first tested whether subjects ate all three food types. Since Bimbo refused to eat apples at the beginning of the data collection (although this food type is desirable for the other subjects and according to the animal keepers he normally liked it), we added a fourth food type (rusk), to confirm that Bimbo´s most prefered food was chosen over the third prefered food in a minimum of 80% of binary choices. Therefore he received additional preference tests before (Food preference test 2, see Table S2) and during the testing phase (Food preference test 3) to ensure that the preference for banana-pellet over grape remained stable over time. Taken together, food preferences of all subjects remained stable (see Table S2).

Procedure

Equally sized food items of different food quality were placed simultaneously onto the sliding table (ca. 1x0.5m) in front of the ape. As the experimenter (IL) moved the table towards the wire mesh, subjects signalled their choice by pointing with the finger towards the more preferred food item. Subjects were given the respective piece of food directly in their mouth or hand, while the other food item was removed immediately. Subjects received four sessions of 12 trials, so that all possible side- and food-combinations were tested 16 times.

Results Qualitative preference test

The orangutans chose their MPF significantly more often than their TPF (Paired Wilcoxon test: $Z=3.416$, $p<0.001$; mean choice MPF over TPF= 97.77%; for details see table S2).

S2 Table Results of the preference tests in percent (%) including all combinations (a=apple, g=grape, p=banana pellet, r=rusk; *TPF*= third preferred food, *MPF*= most preferred food). Preference test 1 was conducted before subjects entered the test, Preference test 4 was conducted after all subjects had received all test trials. Preference test 2 &3 was only conducted with Bimbo due to reasons explained above.

Preference test 1		Combination 1		Combination 2		Combination 3		**Summary**	
Subjects	*Hierarchy*	*apple*	*grape*	*grape*	*pellet*	*apple*	*pellet*	**TPF**	**MPF**
Pini	a < g < p	12,5	87,5	0	100	0	100	0	100

Raja	a < g < p	0	100	12,5	87,5	6,25	93,75	6,25	93,75
Dokana	a < g < p	6,25	93,75	18,75	81,25	0	100	0	100
Padana	a < g < p	6,25	93,75	0	100	0	100	0	100
Suaq	a < g = p	6,25	93,75	50	50	6,25	93,75	6,25	93,75
Bimbo	a < g < p	0	100	25	75	0	100	0	100
Food preference test 2		*rusk*	*grape*	*grape*	*pellet*	*rusk*	*pellet*		
Bimbo	g < r < p	75	25	6,25	93,75	6,25	93,75	6,25	93,75
Food preference test 3				*grape*	*pellet*				
Bimbo	g < p			6,25	93,75			6,25	93,75
Food preference test 4				*grape*	*pellet*	*apple*	*pellet*		
Pini	a < p					0	100	0	100
Raja	a < p					0	100	0	100
Dokana	a < p					6,25	93,75	6,25	93,75
Padana	a < p					0	100	0	100
Suaq	a < p					0	100	0	100
Bimbo	g < p			0	100			0	100
