# Supplementary material for: Orangutans (Pongo abelii) make flexible decisions relative to reward quality and tool functionality in a multi-dimensional tool-use task
Source: PLoS One. 2019 Feb 13;14(2):e0211031. doi: 10.1371/journal.pone.0211031 (PMC6374006; doi:10.1371/journal.pone.0211031)
Supplement: S3 Table — Binomial probabilities: * = p<0.05 (10/12 correct), ** = p<0.01 (11/12 correct); *** = p<0.001 (12/12 correct). (PDF) [file pone.0211031.s003.pdf]

S3 Table Number of correct trials out of a total of 12 trials for each condition in the *TST* for each individual. Binomial probabilities: *= p<0.05 (10/12 correct), **= p<0.01 (11/12 correct); ***= p<0.001 (12/12 correct).

-	TST (Tool selection test)		
	Session 1 + Session 2		
Name	Stick-Apparatus (12 trials)	Ball-Apparatus (12 trials)	Sessions until criterion (minimum: two sessions)
Pini	10*	10*	2
Raja	10*	11**	2
Dokana	12***	4	4
Padana	11**	12***	2
Suaq	9	7	7
Bimbo	10*	8	5
