# Supplementary material for: Orangutans (Pongo abelii) make flexible decisions relative to reward quality and tool functionality in a multi-dimensional tool-use task
Source: PLoS One. 2019 Feb 13;14(2):e0211031. doi: 10.1371/journal.pone.0211031 (PMC6374006; doi:10.1371/journal.pone.0211031)
Supplement: S4 Table — Binomial probabilities: * = p<0.05 (10/12 correct), ** = p<0.01 (11/12 correct); *** = p<0.001 (12/12 correct). (PDF) [file pone.0211031.s004.pdf]

Table S4 Number of correct trials out of a total of 12 trials for each condition in the *QAT* for each individual. Binomial probabilities: *= p<0.05 (10/12 correct), **= p<0.01 (11/12 correct); ***= p<0.001 (12/12 correct).

Name	Quality allocation test (QAT)			
	Session 1 - Session 4			
	Stick-Apparatus		Ball-Apparatus	
	MPF inside (12 trials)	MPF outside (12 trials)	MPF inside (12 trials)	MPF outside (12 trials)
Pini	12***	12***	12***	12***
Raja	10*	12***	11**	11**
Dokana	12***	11**	11**	11**
Padana	12***	12***	12***	12***
Suaq	11**	12***	11**	10*
Bimbo	0	12***	0	11**
