# Supplementary material for: Orangutans (Pongo abelii) make flexible decisions relative to reward quality and tool functionality in a multi-dimensional tool-use task
Source: PLoS One. 2019 Feb 13;14(2):e0211031. doi: 10.1371/journal.pone.0211031 (PMC6374006; doi:10.1371/journal.pone.0211031)
Supplement: S5 Table — Binomial probabilities: * = p<0.05 (10/12 correct), ** = p<0.01 (11/12 correct); *** = p<0.001 (12/12 correct). (PDF) [file pone.0211031.s005.pdf]

S5 Table Number of correct trials out of a total of 12 trials for each condition in the *MT* for each individual. Binomial probabilities: *= p<0.05 (10/12 correct), **= p<0.01 (11/12 correct); ***= p<0.001 (12/12 correct).

Name	Motivation Test (MT)			
	Session 1 + Session 2			
	Stick-Apparatus		Ball-Apparatus	
	Tool (12 trials)	Food (12 trials)	Tool (12 trials)	Food (12 trials)
Pini	12***	0	12***	0
Raja	11**	1	10*	2
Dokana	2	10*	2	10*
Padana	1	11*	2	10*
Suaq	2	10*	1	11**
Bimbo	1	11**	0	12***
