# Supplementary material for: Orangutans (Pongo abelii) make flexible decisions relative to reward quality and tool functionality in a multi-dimensional tool-use task
Source: PLoS One. 2019 Feb 13;14(2):e0211031. doi: 10.1371/journal.pone.0211031 (PMC6374006; doi:10.1371/journal.pone.0211031)
Supplement: S6 Table — Binomial probabilities: * = p<0.05 (10/12 correct), ** = p<0.01 (11/12 correct); *** = p<0.001 (12/12 correct). (PDF) [file pone.0211031.s006.pdf]

S6 Table Number of correct trials out of a total of 12 trials for each condition in the *TFT* for each individual. Binomial probabilities: *= p<0.05 (10/12 correct), **= p<0.01 (11/12 correct); ***= p<0.001 (12/12 correct).

Name	Tool functionality test (TFT)			
	Session 1 - Session 4			
	Stick-Apparatus		Ball-Apparatus	
	Functional vs TPF (12 trials)	Non-functional vs TPF (12 trials)	Functional vs TPF (12 trials)	Non-functional vs TPF (12 trials)
Pini	12***	0	12***	0
Raja	12***	10*	12***	5
Dokana	12***	6	11**	4
Padana	12***	4	12***	6
Suaq	11**	6	12***	5
Bimbo	3	10*	5	9
