# Supplementary material for: Orangutans (Pongo abelii) make flexible decisions relative to reward quality and tool functionality in a multi-dimensional tool-use task
Source: PLoS One. 2019 Feb 13;14(2):e0211031. doi: 10.1371/journal.pone.0211031 (PMC6374006; doi:10.1371/journal.pone.0211031)
Supplement: S8 Table — (PDF) [file pone.0211031.s008.pdf]

S8 Table Results of the paired Wilcoxon tests for subjects´ performance in the ball- and stick-apparatus condition for each condition and for each test (n=6).

	T^+	p (exact, 2-tailed)
TST	3	0,250
QAT MPF inside	1,5	1,000
QAT MPF outside	0	0,250
MT	2,5	0,625
TFT tool functional	4,5	0,750
TFT tool non-functional	3,5	0,375
TSQAT	0	0,125
