# Supplementary material for: Orangutans (Pongo abelii) make flexible decisions relative to reward quality and tool functionality in a multi-dimensional tool-use task
Source: PLoS One. 2019 Feb 13;14(2):e0211031. doi: 10.1371/journal.pone.0211031 (PMC6374006; doi:10.1371/journal.pone.0211031)
Supplement: S9 Table — (PDF) [file pone.0211031.s009.pdf]

Table S9: Results of the paired Wilcoxon tests for the first and last six trials of each condition for each test (n=6).

	T+	p (exact, 2-tailed)
TST Stick-apparatus	10	0.125
TST Ball-apparatus	10	0.625
QAT Stick-app., MPF inside	2	1.000
QAT Stick-app., MPF outside	1	1.000
QAT Ball-app., MPF inside	6	0.250
QAT Ball-app., MPF outside	4	0.750
TFT Stick-app., tool functional	3	0.500
TFT Stick-app., tool non-functional	15	0.063
TFT Ball-app., tool functional	3	0.500
TFT Ball-app., tool non-functional	15	0.063
TSQAT, MPF in Stick-apparatus	5	0.500
TSQAT, MPF in Ball-apparatus	15	0.063
